# Reduced Glutathione in Modulation of Salt Stress on Sour Passion Fruit Production and Quality

**DOI:** 10.3390/plants14142149

**Published:** 2025-07-11

**Authors:** Weslley Bruno Belo de Souza, Geovani Soares de Lima, Lauriane Almeida dos Anjos Soares, Mirandy dos Santos Dias, Brencarla de Medeiros Lima, Larissa Fernanda Souza Santos, Valeska Karolini Nunes Oliveira, Rafaela Aparecida Frazão Torres, Hans Raj Gheyi, Lucyelly Dâmela Araújo Borborema, André Alisson Rodrigues da Silva, Vitor Manoel Bezerra da Silva, Valéria Fernandes de Oliveira Sousa

**Affiliations:** 1 Academic Unit of Agricultural Engineering, Federal University of Campina Grande, Campina Grande 58429-900, CEP, Brazil; weslleybruno56@gmail.com (W.B.B.d.S.); mirandydias@gmail.com (M.d.S.D.); mbrencarla@gmail.com (B.d.M.L.); englarissafss@gmail.com (L.F.S.S.); valeska.karoline2015@gmail.com (V.K.N.O.); rafaelatorres1997@gmail.com (R.A.F.T.); hgheyi@gmail.com (H.R.G.); lucyellyd@gmail.com (L.D.A.B.); vitortn20@gmail.com (V.M.B.d.S.); 2 Academic Unit of Agricultural Sciences, Center for Science and Agrifood Technology, Federal University of Campina Grande, Pombal 58840-000, CEP, Brazil; lauriane.soares@professor.ufcg.edu.br (L.A.d.A.S.); valeriafernandesbds@gmail.com (V.F.d.O.S.); 3 Academic Unit of Agronomy, Universidade Federal do Oeste do Pará, Juruti 68170-000, CEP, Brazil; andrealisson_cgpb@hotmail.com

**Keywords:** *Passiflora edulis* Sims, oxidative stress, salinity

## Abstract

This study evaluated the effects of reduced glutathione (GSH) application on the production and quality of sour passion fruit irrigated with brackish water in the semi-arid region of Paraíba, Brazil. The experiment was conducted in drainage lysimeters under greenhouse conditions at the Center of Technology and Natural Resources of the Federal University of Campina Grande (UFCG). Treatments combined five levels of electrical conductivity of brackish irrigation water (Bw: 0.4, 1.2, 2.0, 2.8, and 3.6 dS m^−1^) and four GSH concentrations (0, 40, 80, and 120 mg L^−1^), arranged in a randomized block design with three replicates. Salinity levels above 0.4 dS m^−1^ negatively affected fruit production and post-harvest quality of ‘BRS GA1’ sour passion fruit. Foliar application of 120 mg L^−1^ GSH increased fruit yield, while 74 mg L^−1^ GSH mitigated salt stress effects on production and pulp chemical quality. The ‘BRS GA1’ cultivar was highly sensitive to salinity, showing a 26.9% yield reduction per unit increase in Bw electrical conductivity above 0.4 dS m^−1^. The results suggest that GSH can alleviate salt stress damage, improving crop productivity and fruit quality under semi-arid conditions.

## 1. Introduction

Fruit farming stands out as one of the most dynamic agricultural activities, playing a vital role in job creation and income generation, through both fresh fruit production and industrial processing [1,2]. Despite the favorable edaphoclimatic conditions in northeastern Brazil—such as high solar radiation and temperatures—the expansion of fruit cultivation in this region is hindered by challenges such as inadequate irrigation and fertilization management, water scarcity, and high salinity in available water sources [3].

Northeastern Brazil, home to approximately 27 million people, is characterized by high temperatures, elevated evapotranspiration rates, and low rainfall, making it one of the largest semi-arid regions in the world [4]. These climatic conditions underscore the region’s reliance on irrigated agriculture to maintain agricultural production [5].

In this context, irrigation is essential for fruit farming in the semi-arid northeast, where the irregular temporal and spatial distribution of rainfall, coupled with high evaporative demand, result in prolonged periods of water deficit for crops [6]. Additionally, the marginal quality of irrigation water—often characterized by high salinity—poses further constraints, requiring adaptive strategies to ensure the sustainable use of limited water resources [7].

Water and/or soil salinity inhibits plant growth through osmotic and ionic effects, which alter the stability of cell membranes and affect physiological and biochemical functions of plants, leading to reductions in turgor and growth, causing disturbances in water relations, reducing the absorption of water and nutrients, decreasing photosynthesis, protein synthesis, enzyme activities, cellular capacity, and increasing oxidative stress [8,9,10].

To avoid oxidative damage, plants have a complex antioxidant defense system composed of enzymatic and non-enzymatic components, capable of neutralizing the toxicity of reactive oxygen species (ROS). The enzymatic mechanism of detoxification involves superoxide dismutase (SOD), catalase (CAT), ascorbate peroxidase (APX), and glutathione reductase (GR) [11,12].

Glutathione reductase is an enzyme responsible for catalyzing the reduction of oxidized glutathione (GSSG) to glutathione (GSH) by the oxidation of reduced coenzyme II (NADPH) [13]. GSH, in turn, is an important molecule within the cellular system, maintaining the intracellular glutathione pool mainly in the reduced state, acting as an antioxidant, being able to eliminate ROS, even participating in the ascorbate glutathione cycle [14], and contributing as an alternative and more efficient detoxification mechanism against H_2_O_2_ generated in the chloroplast and cytosol [15]. GSH participates in the degradation of H_2_O_2_ through a reaction catalyzed by the enzyme glutathione peroxidase. It participates in the ascorbate–glutathione cycle, along with ascorbic acid (AsA), operating on the chloroplast, plastid, mitochondria, and peroxisomes, fighting the overproduction of ROS [16].

Among the fruit crops, sour passion fruit (*Passiflora edulis* Sims) stands out, a tropical fruit plant of great socioeconomic importance, especially in the northeast region, and has stood out mainly among small and medium-sized producers [17]. Its fruits are mainly intended for fresh consumption or, after processing, for the production of juices, sweets, ice cream, among others [18]. In addition to the fruit, different parts of the plant contain phytoconstituents with medicinal properties that can be used [19,20,21].

However, salinity threatens its productivity, necessitating strategies to enhance tolerance. While foliar GSH application has shown promise in alleviating abiotic stress in other crops, its efficacy in improving passion fruit yield and post-harvest quality under brackish water irrigation remains underexplored.

We hypothesized that foliar application of reduced GSH mitigates salt-induced oxidative stress in sour passion fruit by enhancing antioxidant activity, thereby improving fruit yield, physiological performance, and post-harvest quality despite irrigation with brackish water. This study evaluated the effects of foliar GSH on the production and post-harvest quality of sour passion fruit under brackish water irrigation.

## 2. Results

A significant interaction was observed between the levels of electrical conductivity of brackish water Bw and glutathione GSH concentrations (Bw × GSH) on the production per plant (PROD), pulp volume (PV), and pulp yield (PY) of ‘BRS GA1’ sour passion fruit (Table 1), indicating that both factors jointly influenced these variables. In contrast, the total number of fruits (TNFs) was significantly affected by each factor independently, while the average fruit weight (AFW) was influenced solely by the electrical conductivity of the brackish water at 155 days after transplanting (DAT).

**Figure 1:**
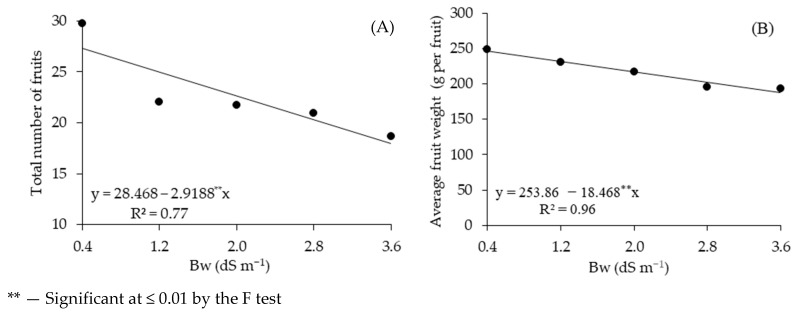
Total number of fruits—TNFs (**A**) and average fruit weight—AFW (**B**) of sour passion fruit as a function of electrical conductivity of brackish irrigation water Bw, observed during the harvest period from 99 to 150 days after transplanting.

Water salinity reduced the total number of fruits of ‘BRS GA1’ sour passion fruit (Figure 1A) by 10.25% per unit increment in Bw, resulting a decrease of 34.21% in the TNFs of plants irrigated with Bw of 3.6 dS m^−1^ compared to those subjected to water salinity of 0.4 dS m^−1^. Similar to TNFs, the average fruit weight was negatively affected by irrigation water salinity (Figure 1B), showing a decrease of 7.27% per unit increment in Bw. Thus, plants irrigated with Bw of 3.6 dS m^−1^ showed a loss of 59.1 g per fruit (23.97%) in AFW compared to those cultivated under water salinity of 0.4 dS m^−1^.

**Figure 2:**
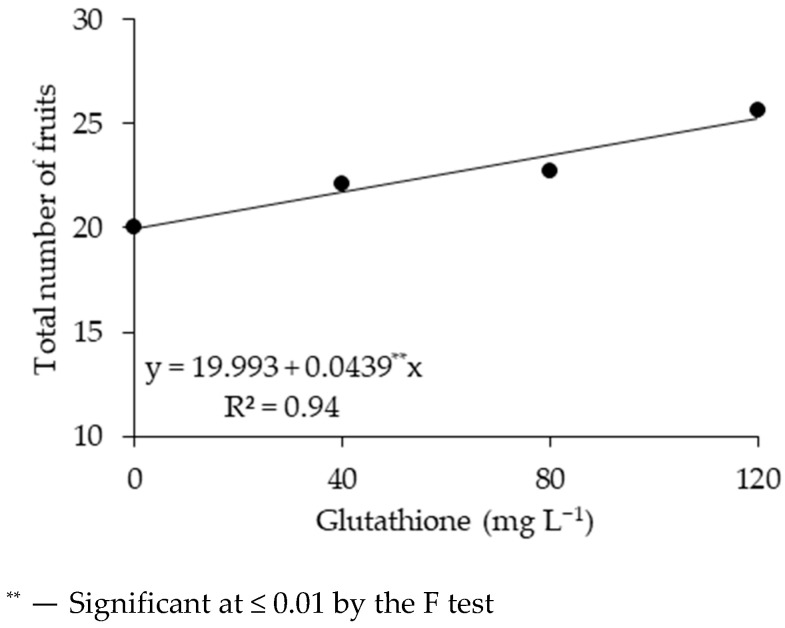
Total number of fruits—TNFs of sour passion fruit as a function of concentrations of reduced glutathione, observed during the harvest period from 99 to 150 days after transplanting.

Foliar application of reduced glutathione promoted a linear increase in the total number of sour passion fruits (Figure 2), with an increase of 26.34% in the TNFs of plants that received 120 mg L^−1^ of GSH compared to the control (0 mg L^−1^).

**Figure 3:**
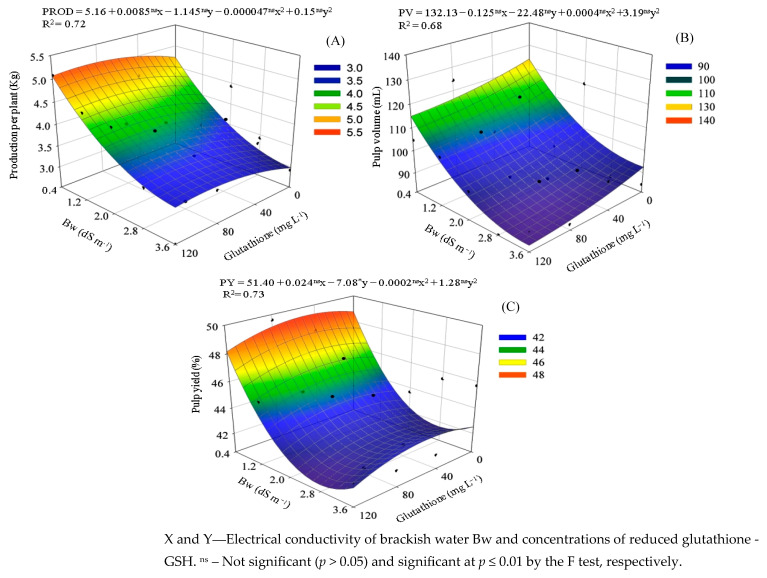
Production per plant—PROD (**A**), pulp volume—PV (**B**), and pulp yield—PY (**C**) of sour passion fruit as a function of the interaction between the electrical conductivity of brackish irrigation water Bw and the concentrations of reduced glutathione observed during the harvest period from 99 to 150 days after transplanting.

The production per plant of sour passion fruit was also negatively affected by the increase in water salinity (Figure 3A), resulting in a lower value (2.98 kg per plant), with a loss of 36.99% in PROD compared to plants cultivated under the lowest salinity level (4.73 kg per plant). GSH application contributed to increasing the PROD of sour passion fruit, especially at the concentration of 90 mg L^−1^, which established the maximum estimated value in plants irrigated with Bw of 0.4 dS m^−1^ (5.11 kg per plant), resulting in an increase of 8.13% compared to plants without application of glutathione. In plants irrigated with water of 3.6 dS m^−1^, the concentration of 90 mg L^−1^ contributed to increase the PROD by 12.89% when compared to plants without GSH application.

Irrigation with 0.4 dS m^−1^ water resulted in the highest pulp volume (PV) (123.64 mL); in contrast, the use of 3.6 dS m^−1^ water reduced PV to 92.54 mL, representing a 25.15% decrease (Figure 3B). Furthermore, the application of glutathione (120 mg L^−1^) to plants irrigated with 3.6 dS m^−1^ water led to the lowest PV value (83.30 mL), corresponding to an additional reduction of 9.98% compared to plants grown under same salinity level without glutathione application. Thus, when relating these data to the values obtained for pulp weight, it was possible to notice a densification resulting from the application of glutathione, which reinforces the idea of translocation of photoassimilates during fruit filling.

Irrigation with water at 3.6 dS m^1^ reduced pulp yield (PY) by 12.86% compared to 0.4 dS m^−1^ water (Figure 3C). However, foliar application of GSH (60 mg L^−1^) mitigated this reduction, increasing PY to 49.49% under low salinity (0.4 dS m^−1^)—a gain of 1.47% compared to untreated plants at the same salinity level. A similar pattern was observed under high salinity (3.6 dS m^−1^), where glutathione contributed to maintaining PY, although absolute values remained lower due to the effects of salt stress.

There was a significant effect of the interaction between the factors (SL × GSH) on hydrogen potential (pH), soluble solids (SSs), titratable acidity (TA), and soluble solids/titratable acidity (SSs/TA) ratio of ‘BRS GA1’ sour passion fruit pulp (Table 2).

**Figure 4:**
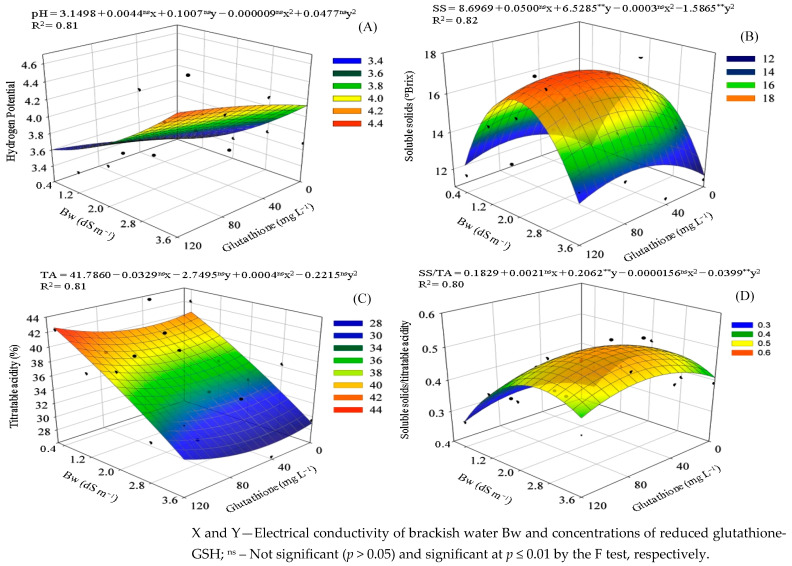
Hydrogen potential—pH (**A**), soluble solids—SSs (**B**), titratable acidity—TA (**C**), and soluble solids/titratable acidity ratio—SSs/TA (**D**) in the pulp of sour passion fruit as a function of the interaction between electrical conductivity of brackish irrigation water Bw and concentrations of reduced glutathione.

The pH of the sour passion fruit pulp increased significantly with rising irrigation water salinity (Figure 4A). In plants irrigated with water at 0.4 dS m^−1^, the pulp pH was 3.20, increasing to 4.13 at 3.6 dS m^−1^—an increase of 29.06%. The application of GSH further intensified this effect, with the highest pH value (4.53) observed in plants treated with 120 mg L^−1^ of GSH and irrigated with 3.6 dS m^−1^ water. This value was 41.56% higher than that of untreated plants at 0.4 dS m^−1^ and 9.68% higher than untreated plants at 3.6 dS m^−1^, indicating that application of GSH enhanced the alkalinizing effect of salinity.

Irrigation with water at 0.4 dS m^−1^ resulted in the lowest soluble solids (SSs) content (11.05 °Brix), while increasing the salinity to 2.1 dS m^−1^ raised this value by 39.46%, reaching 15.41 °Brix (Figure 4B). However, at a salinity of 3.6 dS m^−1^, the SSs content declined to 11.69 °Brix. The foliar application of GSH (83 mg L^−1^) to plants irrigated with 2.1 dS m^−1^ water further enhanced the SSs content, reaching a maximum of 17.49 °Brix—representing increases of 58.28% and 18.82% compared to plants without GSH application at salinities of 0.4 and 2.1 dS m^−1^, respectively. Even under the highest salinity level (3.6 dS m^−1^), GSH application increased SSs to 13.72 °Brix, which was 17.40% higher than the control without GSH, demonstrating its positive effect even under severe saline stress.

The titratable acidity (TA) of sour passion fruit was significantly influenced by the salinity of the irrigation water (Figure 4C). In plants without glutathione application, increasing water salinity from 0.4 to 3.6 dS m^−1^ reduced TA by 28.62%, highlighting the detrimental effect of salt stress on fruit acidity. The application of GSH modified this response. The highest TA value (42.46%) was recorded in plants treated with the combination of 120 mg L^−1^ of GSH and irrigation with 0.4 dS m^−1^ water, representing an increase of 4.46% compared to untreated plants at the same salinity level. Conversely, the lowest TA value (28.34%) was observed in plants treated with 41 mg L^−1^ of GSH under 3.6 dS m^−1^ irrigation water, which was 2.33% lower than that of untreated plants at the same salinity and 33.26% lower than the maximum TA value obtained under optimal conditions.

The soluble solids/titratable acidity (SSs/TA) ratio was significantly affected by the treatments evaluated (Figure 4D). Irrigation with water at 0.4 dS m^−1^ resulted in the lowest SSs/TA ratio (0.259), while increasing salinity to 2.6 dS m^−1^ raised the ratio to 0.449, representing an increase of 73.37%. The application of GSH further enhanced this response, with the highest ratio value (0.520) observed at a concentration of 67 mg L^−1^ under irrigation with 2.6 dS m^−1^ water. This value was 100.77% higher than the control (0.4 dS m^−1^ without GSH) and 15.81% higher than the treatment without GSH at the same salinity level. These findings suggest that both saline stress and GSH application acted synergistically to increase the SSs/TA ratio, indicating a combined influence on fruit ripening and quality.

There was a significant effect of the interaction between the factors (SL × GSH) on the ascorbic acid contents and on all the sugar variables in the pulp of ‘BRS GA1’ sour passion fruit (Table 3). The water consumption of sour passion fruit plants was significantly affected by water salinity levels.

**Figure 5:**
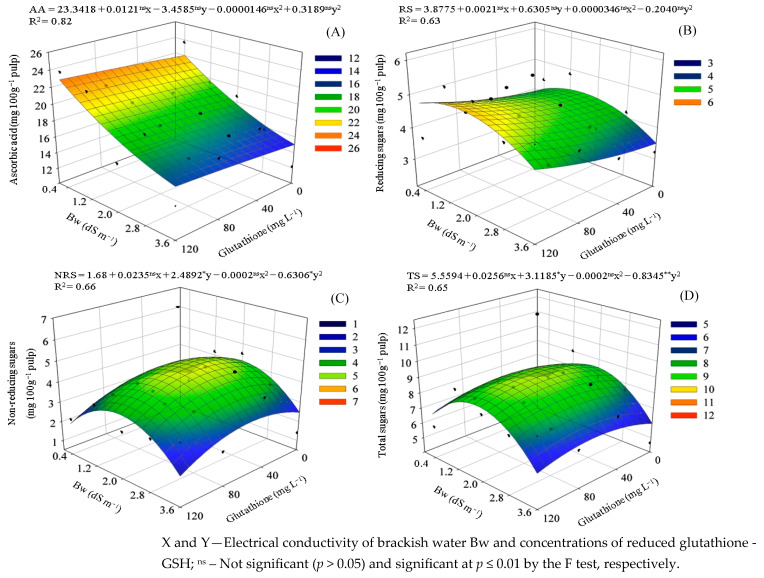
Ascorbic acid—AA (**A**), reducing sugars—RSs (**B**), non-reducing sugars—NRSs (**C**), and total sugars—TSs (**D**) in the pulp of passion fruit as a function of the interaction between the electrical conductivity of brackish irrigation water Bw and the concentrations of reduced glutathione.

Increasing irrigation water salinity led to a reduction in ascorbic acid (AA) content in the pulp (Figure 5A), with a 31.76% decrease observed as salinity increased from 0.4 dS m^−1^ (22.01 mg 100 g^−1^ pulp) to 3.6 dS m^−1^ (15.02 mg 100 g^−1^ pulp). However, foliar application of GSH (120 mg L^−1^) to plants irrigated with 0.4 dS m^−1^ water resulted in the highest AA content (23.25 mg 100 g^−1^ of pulp), representing 5.64% increase compared to untreated plants at the same salinity and a 54.76% increase compared to the lowest value recorded in plants irrigated with 3.6 dS m^−1^ water without GSH.

The treatment with high-salinity water (3.6 dS m^−1^) resulted in the lowest reducing sugar content (3.50 mg 100 g^−1^ pulp), representing a 14.49% decrease compared to irrigation with 0.4 dS m^−1^ water (4.10 mg 100 g^−1^ pulp) (Figure 5B). The application of GSH at 120 mg L^−1^ promoted a significant increase in the reducing sugar content, raising it from 4.36 to 5.11 mg 100 g^−1^ pulp (a 17.19% increase) under irrigation with 1.5 dS m^−1^ water. This was the highest value recorded for the variable. Notably, even under high salinity (3.6 dS m^−1^), application of GSH at a concentration of 120 mg L^−1^ increased reducing sugar levels by 21.41% compared to untreated plants under the same condition. These results demonstrate the effectiveness of glutathione in mitigating the negative impact of salt stress on sugar accumulation in sour passion fruit pulp.

Non-reducing sugar (NRSs) content in sour passion fruit pulp was influenced by both Bw and GSH application (Figure 5C). Increasing salinity up to 2.0 dS m^−1^ significantly promoted accumulation, with a peak value of 4.14 mg 100 g^−1^ of pulp, representing an increase of 60.63% compared to the 0.4 dS m^−1^ treatment (2.57 mg 100 g^−1^ of pulp). Foliar application of GSH at 60 mg L^−1^ further enhanced this effect, raising NRSs content to 4.83 mg 100 g^−1^ of pulp under 2.0 dS m^−1^ water—a 16.69% increase over untreated plants at the same salinity. A similar positive response to glutathione was observed under more severe saline stress (3.6 dS m^−1^), where the same concentration of GSH (60 mg L^−1^) increased NRSs from 2.47 to 3.16 mg 100 g^−1^ of pulp, representing a gain of 28.01%.

Total sugar (TSs) content in sour passion fruit pulp was significantly affected by irrigation water salinity and glutathione (GSH) application (Figure 5D). The salinity level of 1.9 dS m^−1^ promoted the highest accumulation (8.47 mg 100 g^−1^ of pulp), representing a 26.95% increase compared to the 0.4 dS m^−1^ treatment (6.67 mg 100 g^−1^ of pulp). In contrast, the highest salinity level (3.6 dS m^−1^) resulted in the lowest TSs content (5.97 mg 100 g^−1^ of pulp), with reductions of 10.49% and 29.51% relative to the 0.4 and 1.9 dS m^−1^ treatments, respectively. Foliar application of GSH at 64 mg L^−1^ had positive effects at both salinity levels. In plants irrigated with 1.9 dS m^−1^ water, GSH increased TSs content by 9.67% compared to untreated plants under the same salinity. Under severe saline stress (3.6 dS m^−1^), GSH application led to an even greater increase of 13.72% compared to the untreated control, demonstrating its effectiveness in mitigating salt-induced reductions in sugar accumulation.

**Figure 6:**
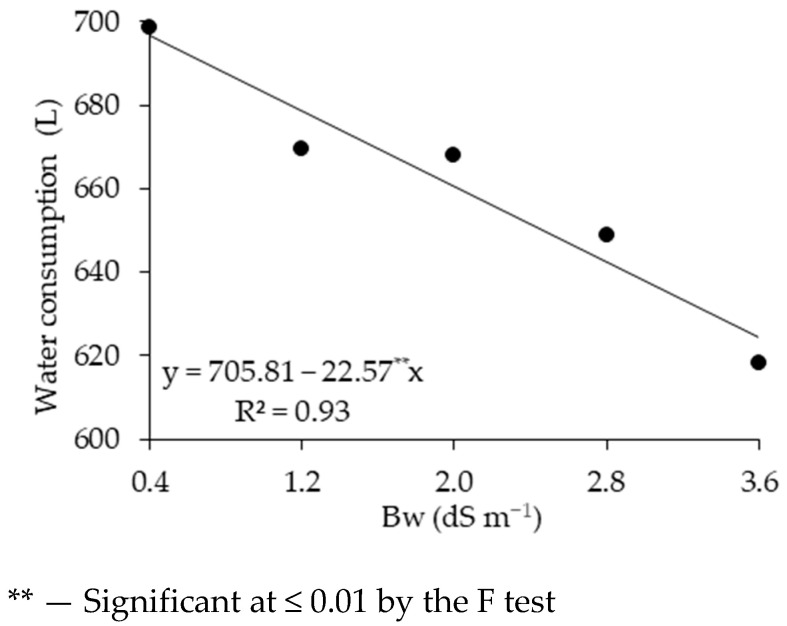
Water consumption of sour passion fruit as a function of brackish irrigation water Bw salinity up to 155 days after transplanting.

The water consumption (WC) of sour passion fruit decreased significantly due to the increase in the electrical conductivity of irrigation water (Figure 6), leading to a reduction of 72.22 L in plants irrigated with water of 3.6 dS m^−1^ compared to those subjected to 0.4 dS m^−1^, representing a decrease of 10.36%.

**Figure 7:**
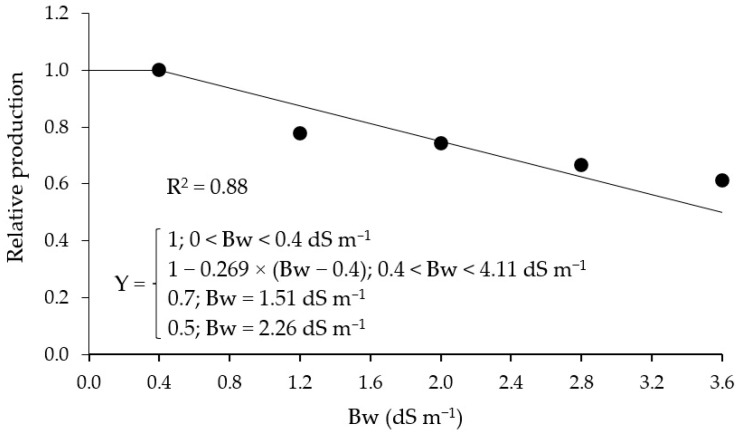
Relative production of sour passion fruit as a function of the electrical conductivity of brackish irrigation water Bw, described by the plateau followed by linear decrease model [22].

The irrigation water salinity threshold obtained by the plateau followed by linear decrease model for ‘BRS GA1’ sour passion fruit was 0.4 dS m^−1^, with a reduction of 26.9% per unit increment of salinity in Bw. Based on the equation, irrigation with water of 1.51 dS m^−1^ results in a relative production of 70%, and irrigation with water of 2.26 dS m^−1^ allows a relative production of 50% (Figure 7). Thus, based on the observed relative production and reductions with per unit increase in water salinity, the passion fruit cultivar ‘BRS GA1’ is classified as sensitive to salt stress.

## 3. Discussion

Salt stress exerted profound negative effects on sour passion fruit production through multiple physiological disruptions. Our findings demonstrate that high salinity irrigation water (Bw) significantly reduced both the number of fruits and average weight, consistent with previous reports [23,24]. For instance, an increase in Bw salinity from 0.35 to 4.0 dS m^−1^ resulted in an 11.80% reduction (13.25 fruits per plant) in fruit number [23]. Similarly, Ref. [24] evaluated the production and post-harvest quality of sour passion fruit irrigated with saline waters and exogenous application of H_2_O_2_ and found a 42.30% reduction in the NFs of plants grown under Bw of 3.0 dS m^−1^ compared to those subjected to the lowest water salinity (0.6 dS m^−1^), without hydrogen peroxide application.

The reduction in number of fruits (NFs) under salt stress can be attributed to the negative impact of salinity on fruit production, which may be associated with both a decrease in the number of productive branches and a reduction in internode length and floral bud development [25]. The reduction in fruit weight is attributed to physiological alterations that hinder photoassimilate translocation, as plants prioritize osmotic adjustment and ion exclusion under stress [9]. This metabolic reallocation limits energy available for fruit development, exacerbating production losses. This reflects the net accumulation of water in the fruit, since it is influenced by the entry and exit of water as well as by photosynthetic products [26].

The decrease in production due to salt stress can be explained by limitations in gas exchange, which directly interferes with the allocation of photoassimilates, causing the plant to use energy to restrict the absorption of Na^+^ and the biosynthesis of osmolytes to reduce the osmotic potential under salt stress, which leads to a reduction in production [27,28].

However, the use of glutathione improved total fruit production. Reduced glutathione (GSH) plays a central role as one of the main non-enzymatic components of the antioxidant system, preventing the formation of free radicals, either through sequestration or degradation, thus preventing potential cell damage [16,29]. Therefore, high levels of cellular GSH can establish the maintenance of the flowering process, avoiding disturbances in the formation of the pollen tube normally associated with a reduction in the number of passion fruits [30]. On the other hand, the beneficial effect of GSH is associated with its role in maintaining the redox balance in cells, neutralizing ROS and contributing to cell protection, and, perhaps, to the formation and filling of the fruit [14].

The low influence of GSH on the increase in PY may be related to the balance between the production of pulp and peel by the fruit, while the reductions observed in plants under salt stress reflect their photosynthetic losses, leading to changes in the fruit filling process, a behavior that has already been observed by [31,32] in sour passion fruit.

Water salinity elevates pulp pH and soluble solids (SSs). Similar results were found by [26] in studying the use of brackish water and potassium fertilization on the post-harvest quality of passion fruit; the highest SSs content (14.90 °Brix) and pH (2.49) were observed in fruits from plants irrigated with 2.7 dS m^−1^ water. This fact demonstrates that salinity is the main factor for increasing the pH of sour passion fruit pulp, which can be explained by the increase in the translocation of photoassimilates, given the reductions in production, reducing fruit acidity [33].

The benefits of glutathione are associated with the maintenance of the plant’s antioxidant activity, enhancing the regulation of ROS production [34], which are the major limiting factors for solute translocation to fruits. This is particularly important under stress conditions, where plants tend to allocate more resources toward metabolic regulation [35]. However, even the lowest SSs value (11.05 °Brix) is within the range recommended by normative instruction No. 37, of October 1, 2018, for fruits of sour passion fruit, which establishes a minimum pH of 2.7, °Brix of 11, and total acidity greater than 2.5 [36].

These results demonstrate that GSH caused a slight change in the acidity of sour passion fruit pulp, with the extremes being determined by the salinity level. This is consistent with the increase in SSs content and pH due to the increases in irrigation water salinity. However, the values of total acidity are high, indicating that the pulp has a high concentration of free protons and undissociated acids, desirable characteristics for the industry since they inhibit metabolic pathways and prevent the propagation of microorganisms [37].

The increase in the SSs/TA ratio reinforces the idea of investment of solutes in sink organs and, despite affecting carbon fixation, the reduction in the number and weight of the fruits compensates for photosynthetic losses and increases the chemical quality of the fruit [38]. This is different from the effects of glutathione, which through metabolic regulation promote improvements in photosynthesis, increasing the support to fruit filling [39].

In the present study, the highest AA contents in the fruits were obtained when the plants received the application of 120 mg L^−1^ of GSH and were irrigated with water of 0.4 dS m^−1^. This behavior may be related to the investment of AA in the leaves, since it acts in the activity of ascorbate reductase and as an electron donor in the photochemical phase of photosynthesis, increasing the efficiency in the control of ROS [40].

The increase in GSH concentration led to an increase in the levels of reducing sugars in passion fruit pulp at lower and higher salt concentrations. RS contents are related to the synthesis of soluble sugars, which contribute to the stress condition signaling process [41]. Thus, at moderate salinity levels, and with the application of glutathione, the increase in RSs may be associated with the regulation of the fruit’s metabolic activity, in addition to contributing to the sweet taste, a characteristic desirable by the fresh fruit market [4,42].

The accumulation of NRSs showed a behavior similar to that of RSs, maintaining a constant relationship in the production of soluble sugars and sugars that require hydrolysis for oxidation, an interesting response to the commercialization process since they are associated with fruit conservation characteristics [43,44].

This response is consistent with the increase in the contents of soluble solids observed in sour passion fruit pulp, considering that the inorganic carbon fixed by photosynthesis is initially converted into carbohydrates in the form of sugars, such as glucose, fructose, sucrose, and starch, which are transported and destined for the most diverse functions in the plant, especially to fruit filling in the fruiting stage [41,45].

The water consumption (WC) of sour passion fruit decreased significantly due to the increase in the electrical conductivity of irrigation water. The reduction in water consumption in plants cultivated under salt stress may be a consequence of water retention due to the accumulation of salts close to the root system, reducing the absorption of water and nutrients by the roots; this justifies the energy expenditure to maintain the plant’s water potential, which results in quantitative and qualitative losses in the fruiting stage [46].

In summary, based on the observed relative production and reductions with per unit increase in water salinity, the passion fruit cultivar studied (‘BRS GA1’) is classified as sensitive to salt stress. Ref. [47], in a study on the salt tolerance (Bw—0.3 to 3.5 dS m^−1^) of sour passion fruit cultivars (‘BRS GA1’, ‘BRS SC1’, and ‘SCS437’), observed that the cultivar ‘BRS GA1’ is sensitive, and verified losses in production above Bw of 0.3 dS m^−1^.

These findings highlight the complex trade-offs between salt tolerance mechanisms and fruit production. The biochemical improvements mediated by GSH, particularly in sugar metabolism and antioxidant capacity, suggest its potential as a complementary strategy for passion fruit cultivation in lower irrigation water salinity. However, the cultivar’s inherent sensitivity to salinity indicates that GSH application should be combined with other approaches (e.g., irrigation management, rootstock selection) for optimal results.

## 4. Materials and Methods

### 4.1. Location of the Experimental Site

This study was conducted from April to December 2023 in a greenhouse at the Center of Technology and Natural Resources, Federal University of Campina Grande (UFCG), Brazil (7°15′18″ S, 35°52′28″ W; 550 m a.s.l). The region features a BSh Köppen climate (hot semi-arid), characterized by irregular rainfall distribution and an annual average precipitation of 802.7 mm [48]. During the experimental period, temperatures ranged from 19.2 °C (minimum) to 27.5 °C (maximum), with mean relative humidity of air of 83%.

### 4.2. Experimental Design and Treatments

The plants were distributed in a randomized block design, in a 5 × 4 factorial arrangement, with three replicates. Treatments consisted of a combination of two factors: five levels of electrical conductivity of brackish irrigation water (Bw) (0.4, 1.2, 2.0, 2.8, and 3.6 dS m^−1^), associated with four concentrations of reduced glutathione (GSH) (0, 40, 80, and 120 mg L^−1^). In each lysimeter, one plant was used per plot, totaling 60 experimental units. The Bw levels were based on studies conducted by evaluating the effects of irrigation with saline waters on cv. BRS GA1 and [45,49] with the sour passion fruit cultivars ‘BRS GA1’, ‘BRS SC1’, and ‘SCS437’. Reduced glutathione (GSH) concentrations were adopted according to the study conducted by Mahmoud, Sadak, and Elhamid [50].

The seeds used in the experiment were obtained from the sour passion fruits of cultivar ‘BRS Gigante Amarelo’ (‘BRS GA1’), a hybrid variety known for its oblong-shaped fruits with a slightly flattened base and apex. The fruits typically weigh between 120 and 350 g, yield approximately 40% pulp, and contain 13 to 15 °Brix of soluble solids. This cultivar is also recognized for its tolerance to anthracnose [51].

### 4.3. Experimental Setup and Conduction

Sowing was carried out by planting 3 seeds in plastic bags with dimensions of 15 × 20 cm, filled with 1.8 kg of soil. After emergence, when the seedlings were about 10 cm tall, thinning was carried out, leaving only one plant per bag. Fertilization with nitrogen, phosphorus, and potassium during the seedling formation phase was carried out according to the recommendations of [52], applying the amounts of 100, 150, and 300 mg kg^−1^ of soil of N, K_2_O, and P_2_O_5_, respectively, split into four equal applications, via irrigation water. Irrigations were carried out daily using water with EC of 0.4 dS m^−1^ during the entire seedling formation period.

Before transplanting the seedlings to the lysimeters, soil samples were collected at 0–20 cm depth and mixed to form a composite sample with the following chemical and physical characteristics: pH= 5.40; OM = 7.62 dag kg^−1^; P = 2.92 mg kg^−1^; K^+^= 0.28 cmol_c_ kg^−1^; Na^+^ = 0.04 cmol_c_ kg^−1^; Ca^2+^ = 1.87 cmol_c_ kg^−1^; Mg^2+^ = 1.70 cmol_c_ kg^−1^; Al^3+^ = 0.20 cmol_c_ kg^−1^; H^+^ = 2.85 cmol_c_ kg^−1^; electrical conductivity of the saturation extract = 0.72 dS m^−1^; cation exchange capacity = 6.94 cmol_c_ kg^−1^; sodium adsorption ratio of the saturation extract = 1.86 mmol L^−1^; exchangeable sodium percentage = 0.57%; sand = 675.2 g kg^−1^; silt = 221.1 g kg^−1^; clay = 103.7 g kg^-1^; moisture 33.42 kPa = 12.94 dag kg^−1^; moisture 1519.5 kPa = 5.32 dag kg^−1^. These characteristics were obtained according to the methodologies recommended in [53]. The pH was determined in a 1:2.5 ratio of soil and water. The soil was classified as Neossolo Regolítico (*Psamments*) [53,54] with sandy loam texture, from the municipality of Lagoa Seca, PB-Brazil.

Before transplanting the seedlings to the lysimeters, the soil moisture content was raised to the level corresponding to the maximum water retention capacity, with water of 0.4 dS m^−1^. At transplanting, the seedlings had an average main stem length of 1.0 m. Recipients with a capacity of 200 L adapted as drainage lysimeters were used. At the lower base of each lysimeter, two 16 mm diameter drains were installed, and a geotextile was placed to prevent clogging of the drains; over the geotextile, a 0.5 kg layer of crushed stone (number. zero) and 250 kg of soil were placed. The drained water was collected in two 2 L plastic bottles placed below each lysimeter to determine plants’ water consumption.

The training system used was the vertical trellis, as described by [9]. Throughout the experiment, tendrils and unwanted branches were eliminated to favor the full development of the crop, according to procedures adopted by [45]. Pollination was carried out manually, until the beginning of harvest, and by carpenter bees (*Xylocopa* spp.), which carried out natural pollination.

During the crop cycle, the irrigation water of the treatment with the lowest level of electrical conductivity (0.4 dS m^−1^) came from the municipal supply system of Campina Grande, PB. The other Bw levels were obtained by the dissolution of the salts NaCl, CaCl_2_.2H_2_O, and MgCl_2_.6H_2_O, in the equivalent proportion of 7:2:1 [55], respectively, in the local supply water of Campina Grande, PB, considering the relationship between Bw and salt concentration [56].

Irrigation with brackish water began 72 h after the first application of reduced glutathione. Irrigation was carried out daily, applying to each container the volume corresponding to that obtained by the water balance (volume applied in previous irrigation volume drained). A leaching fraction of 0.15 was applied every 15 days, aiming to reduce the accumulation of salts in the root zone of the plants.

After transplanting the seedlings in the lysimeters, fertilization was carried out according to the recommendation of [57]. Fertilization with nitrogen, phosphorus, and potassium began at 15 days after transplanting (DAT) and was applied fortnightly via fertigation. Urea (45% N), monoammonium phosphate (60% P_2_O_5_ and 12% N), and potassium sulfate (51.5% K_2_O and 17% S) were used as sources of nitrogen, phosphorus, and potassium, respectively. A quantity of 65 g of N per plant was used in the vegetative stage, and 160 g of N per plant was applied in the flowering and fruiting stages. For potassium, 65 g of K_2_O per plant was applied in the crop formation phase (vegetative stage), and 280 g of K_2_O per plant was applied in the flowering and fruiting stages. For phosphorus, 50 g of P_2_O_5_ per plant was applied in the cycle.

The micronutrients were applied every 10 days throughout the cycle, using a knapsack sprayer containing a solution of 1.0 g L^−1^ of Dripsol Micro Rexene Equilíbrio^®^ (1.2% Mg, 0.85% B, 3.4% Fe, 4.2% Zn, 3.2% Mn, 0.5% Cu, and 0.06% Mo). Phytosanitary control as well as corrective spraying were carried out as necessary to control the possible emergence of pests and diseases. Phytosanitary control was carried out using Cercobin^®^ and Folicur^®^ as fungicides, Lannate^®^, Battus^®^, and Evidence^®^ with insecticidal action, and Vertimec^®^ with acaricidal, insecticidal, and nematicidal action.

The solutions of reduced glutathione were prepared dissolving Reduced L-Glutathione (GSH) in distilled water, according to each treatment, and the nonionic adhesive spreader Wil Fix^®^ was added at a concentration of 0.5 mL L^−1^ in all treatments, in order to reduce the flow of the product, fixing it for a longer time on the leaf blade, promoting greater absorption by the leaves. Plants of the control treatment (0 mg L^−1^) received only distilled water with the adhesive spreader.

GSH applications began at 40 DAT and, later, the applications were carried out at 15-day intervals until the flowering stage. The applications were carried out from 4:30 p.m., and each plant was isolated using TNT (Non-woven fabric) curtains to avoid drift of the solution between plants of different treatments. During the experiment, an average of 8 L of GSH solution was used per treatment.

### 4.4. Traits Analyzed

The production of sour passion fruit was evaluated based on the number of fruits per plant (TNFs), obtained by counting all fruits produced per plant, and the production (PROD) per plant (kg), determined by weighing the harvested fruits on a semi-analytical scale with a precision of 3.0 g. Average fruit weight (AFW) was calculated by the ratio between the total production per plant and the total number of fruits. Internal attributes (pulp volume, pulp weight, and pulp yield) were also determined. The harvest point was based on the color of the fruits, defined by the change from green to yellow. Harvest was carried out continuously from mid-October to early December.

Post-harvest quality was evaluated by determining soluble solids (SSs), hydrogen potential (pH), reducing (RSs) and non-reducing sugars (NRSs), total sugars (TSs), titratable acidity (TA), and ascorbic acid (AA). The pulp samples were separated for further analysis in zip lock plastic bags (10 cm × 15 cm), containing approximately 250 g of pulp per treatment, resulting from the mixture of pulp from random fruits from the plants of each treatment.

Total soluble solids and titratable acidity were determined according to the methodology recommended by Instituto Adolfo Lutz [58], and ascorbic acid contents were determined by the titration method, until the solution acquired a blue color, resulting in a percentage (mg 100 g^−1^ of pulp) [58]. Hydrogen potential (pH) measurements were taken directly using a digital pH meter calibrated with pH 4.0 and 7.0 buffer solutions. Sugar content analysis was performed through the anthrone reaction method, which quantified both reducing and total sugars [59].

Water consumption of sour passion fruit plants was determined using data obtained by the water balance, according to Equation (1):

WC = ∑Va (L) − ∑ Vd (L)(1)

where

∑Va = Sum of the volume applied in all irrigation events (L); and

∑Vd = Sum of the volume drained throughout the cycle (L).

Sour passion fruit tolerance to salt stress was determined based on the relative production per plant, using the plateau followed by linear decrease model of [22]. The model parameters were obtained by minimizing the square of the errors with the Microsoft Excel version 11 Solver tool, according to [60].

### 4.5. Statistical Analysis

The data were first tested for normality using the Shapiro–Wilk test. Subsequently, an analysis of variance (ANOVA) was conducted at 5% (*) and 1% (**) significance levels. Where significant effects were observed, linear and quadratic regression analyses were performed using the SISVAR-ESAL statistical software [61]. The selection of the regression model (linear or quadratic) was based on the significance of the coefficient of determination. For cases where significant interactions between factors were observed, response surfaces were generated using SigmaPlot 12.5 software.

## 5. Conclusions

Irrigation with brackish water above 0.4 dS m^−1^ reduces the production and post-harvest quality of ‘BRS GA1’ sour passion fruit, as this variety is sensitive to salinity. Each unit increase in salinity of brackish water above this threshold leads to a 26.9% decrease in productivity. However, foliar application of reduced glutathione can mitigate the effects of salt stress. When applied at a concentration of 120 mg L^−1^, reduced glutathione increases the number of fruits produced. At a concentration of 74 mg L^−1^, it helps reduce the negative impacts of salinity on both fruit production and pulp quality of ‘BRS GA1’ sour passion fruit. These strategies may serve as viable alternatives to maintain productivity and fruit quality under saline irrigation conditions.

## Figures and Tables

**Table 1:** Summary of the F-test results for total number of fruits (TNFs), production per plant (PROD), average fruit weight (AFW), pulp volume (PV), and pulp yield (PY) of sour passion fruit grown under varying levels of electrical conductivity of brackish water (Bw) and concentrations of reduced glutathione (GSH), observed during the harvest period from 99 to 150 days after transplanting.

Sources of Variation	F-Test				
	TNFs	PROD	AFW	PV	PY
Brackish water (Bw)	**	**	**	**	**
Linear regression	**	**	**	**	**
Quadratic regression	ns	ns	ns	**	**
Reduced glutathione (GSH)	*	ns	ns	**	ns
Linear regression	**	ns	ns	**	ns
Quadratic regression	ns	ns	ns	ns	ns
Interaction (Bw × GSH)	ns	*	ns	**	**
Blocks	ns	ns	ns	ns	ns
CV (%)	12.19	10.95	15.89	8.85	6.76

CV (%)—Coefficient of variation; (*) significant at 0.05 probability level; (**) significant at 0.01 probability level; (ns) not significant.

**Table 2:** Summary of the F-test results for hydrogen potential (pH), soluble solids (SSs), titratable acidity (TA), and soluble solids/titratable acidity (SSs/TA) ratio of ‘BRS GA1’ sour passion fruit grown under varying levels of electrical conductivity of brackish irrigation water (Bw) and concentrations of reduced glutathione (GSH).

Sources of Variation	F-Test			
	pH	SSs	TA	SSs/TA
Brackish water (Bw)	**	**	**	**
Linear regression	**	ns	**	**
Quadratic regression	*	**	ns	**
Reduced glutathione (GSH)	**	**	**	**
Linear regression	**	**	**	*
Quadratic regression	ns	**	**	**
Interaction (Bw × GSH)	**	**	**	**
Blocks	ns	ns	ns	ns
CV (%)	4.80	6.14	3.80	7.44

CV (%)—Coefficient of variation; (*) significant at 0.05 probability level; (**) significant at 0.01 probability level; (ns) not significant.

**Table 3:** Summary of the F-test results for ascorbic acid (AA), reducing sugars (RSs), non-reducing sugars (NRSs), total sugars (TSs) of the pulp, and water consumption (WC) of ‘BRS GA1’ sour passion fruit grown under varying levels of electrical conductivity of brackish irrigation water (Bw) and concentrations of reduced glutathione (GSH).

Sources of Variation	F-Test				
	AA	RSs	NRSs	TSs	WC
Brackish water (Bw)	**	**	**	**	**
Linear regression	**	**	ns	*	**
Quadratic regression	*	**	**	**	ns
Reduced glutathione (GSH)	**	**	**	**	ns
Linear regression	**	**	ns	ns	ns
Quadratic regression	ns	ns	**	**	ns
Interaction (Bw × GSH)	**	**	*	**	ns
Blocks	ns	ns	ns	ns	ns
CV (%)	5.61	8.88	25.96	11.11	5.92

CV (%)—Coefficient of variation; (*) significant at 0.05 probability level; (**) significant at 0.01 probability level; (ns) not significant.

## Data Availability

All data generated and/or analyzed during the present study are available in the manuscript, and the corresponding author has no objection to the availability of data and materials upon reasonable request.
